# *In vitro* biosafety profile evaluation of multipotent mesenchymal stem cells derived from the bone marrow of sarcoma patients

**DOI:** 10.1186/1479-5876-12-95

**Published:** 2014-04-09

**Authors:** Enrico Lucarelli, Chiara Bellotti, Melissa Mantelli, Maria Antonietta Avanzini, Rita Maccario, Francesca Novara, Giulia Arrigo, Orsetta Zuffardi, Monia Zuntini, Martina Pandolfi, Luca Sangiorgi, Daniela Lisini, Davide Donati, Serena Duchi

**Affiliations:** 1 Osteoarticolar Regeneration Laboratory, Rizzoli Orthopaedic Institute, Bologna, Italy; 2 Immunology and Transplantation Laboratory/Cell Factory/ Pediatric Oncohematology, Fondazione IRCCS Policlinico S. Matteo, Pavia, Italy; 3 Department of Molecular Medicine, University of Pavia, Pavia, Italy; 4 Department of Medical Genetics and Skeletal Rare Diseases, Rizzoli Orthopaedic Institute, Bologna, Italy; 5 Cell Therapy Production Unit (UPTC), IRCCS Neurologic Institute C. Besta Foundation, Milano, Italy; 6 Department of Biomedical and Neuromotor Sciences (DIBINEM), Alma Mater Studiorum University of Bologna, Bologna, Italy; 7 Osteoarticular Regeneration Laboratory, 3rd Clinic of Orthopaedics and Traumatology, Rizzoli Orthopaedic Institute, Bologna, Italy

**Keywords:** Ewing sarcoma (EWS), Mesenchymal stem cells (MSCs), Osteosarcoma (OS), Tissue regeneration, Tumorigenic transformation

## Abstract

**Background:**

In osteosarcoma (OS) and most Ewing sarcoma (EWS) patients, the primary tumor originates in the bone. Although tumor resection surgery is commonly used to treat these diseases, it frequently leaves massive bone defects that are particularly difficult to be treated. Due to the therapeutic potential of mesenchymal stem cells (MSCs), OS and EWS patients could benefit from an autologous MSCs-based bone reconstruction. However, safety concerns regarding the *in vitro* expansion of bone marrow-derived MSCs have been raised. To investigate the possible oncogenic potential of MSCs from OS or EWS patients (MSC-SAR) after expansion, this study focused on a biosafety assessment of MSC-SAR obtained after short- and long-term cultivation compared with MSCs from healthy donors (MSC-CTRL).

**Methods:**

We initially characterized the morphology, immunophenotype, and differentiation multipotency of isolated MSC-SAR. MSC-SAR and MSC-CTRL were subsequently expanded under identical culture conditions. Cells at the early (P3/P4) and late (P10) passages were collected for the *in vitro* analyses including: sequencing of genes frequently mutated in OS and EWS, evaluation of telomerase activity, assessment of the gene expression profile and activity of major cancer pathways, cytogenetic analysis on synchronous MSCs, and molecular karyotyping using a comparative genomic hybridization (CGH) array.

**Results:**

MSC-SAR displayed comparable morphology, immunophenotype, proliferation rate, differentiation potential, and telomerase activity to MSC-CTRL. Both cell types displayed signs of senescence in the late stages of culture with no relevant changes in cancer gene expression. However, cytogenetic analysis detected chromosomal anomalies in the early and late stages of MSC-SAR and MSC-CTRL after culture.

**Conclusions:**

Our results demonstrated that the *in vitro* expansion of MSCs does not influence or favor malignant transformation since MSC-SAR were not more prone than MSC-CTRL to deleterious changes during culture. However, the presence of chromosomal aberrations supports rigorous phenotypic, functional and genetic evaluation of the biosafety of MSCs, which is important for clinical applications.

## Background

Bone sarcomas, a heterogeneous group of rare malignant tumors of mesenchymal origin [1], occur primarily in adolescents and young adults. Bone sarcomas are classified genetically into two categories: osteosarcoma (OS) is characterized by complex karyotypes indicative of severe genetic and chromosomal instability [2], while Ewing’s sarcoma (EWS) is characterized by the presence of tumor-specific translocations. OS is the most common primary tumor of the bone and it is usually found at the end of long bones, often around the knee [3]. The etiology of OS is not well understood, as well as a clear link between OS and inherited genetic mutations or specific genetic changes has not been established, although patients with Li-Fraumeni syndrome have a high risk of developing OS by inheriting mutations that silence the p53 tumor suppressor gene (for a comprehensive review see [4]). EWS typically develops in the femur and tibia. The most common mutation associated with EWS involves a translocation of chromosomes 22 and 11 (t (11;22)), which fuses a portion of the *EWSR1* gene with a portion of the *FLI1* gene to create a *EWS/FLI-1* fusion. This is a non-inheritable somatic mutation acquired only in tumor cells during a person’s lifetime [5,6].

Despite considerable improvements in the diagnosis and treatment of OS and EWS, progress in patient survival has remained stagnant for more than two decades [7-9]. Current OS and EWS treatments consist of multiple modalities, traditionally including amputation or limb-sparing surgery, with the goal of complete tumor removal. Adjuvant therapies—such as radiation and chemotherapy—are used selectively in an effort to minimize both local recurrence and distant metastasis of the disease. Tumor resection often causes a massive bone defect that is difficult to treat. Thus, OS and EWS patients could benefit from a mesenchymal stem cell (MSC)-based therapeutic approach to bone reconstruction, alone or in combination with biomaterials to provide a structural support.

Recognition of the regenerative potential of MSCs is one of the most exciting fields in cell-based therapy; their safety and efficacy has been reported in > 250 clinical trials [10]. MSCs are appealing because they can be isolated easily from bone marrow (BM) and several other human tissues, can be expanded *in vitro*, have a high proliferative capacity, lack immunogenicity, display immunomodulatory properties, retain the ability to secrete soluble factors that regulate crucial biological functions, such as proliferation and differentiation over a broad spectrum of target cells [11], and target damaged tissues and tumor sites (for a review see [12,13]). Most importantly, the ability of MSCs to differentiate into several cell lineages makes them ideal for reparative medicine [14-16].

The use of MSCs for clinical applications requires *in vitro* expansion. However, there is concern about the chromosomal stability and biosafety of expanded human MSCs, particularly those derived from sarcoma patients (for updated reviews see [17,18]). Several studies have indicated that murine MSCs acquire chromosomal abnormalities after a few *in vitro* passages and generate OS after the *in vivo* transplantation [19,20]. In contrast, MSCs derived from healthy human donors or patients with Crohn’s disease do not undergo malignant transformation after the *in vitro* expansion [21-26]. Centeno *et al. *[27,28] reported that 227 patients treated for various orthopedic conditions by implanting autologous MSCs that had been expanded *in vitro* using growth factors supplied by a platelet lysate did not experience any evident neoplastic complication with > 2 years of follow-up. Thus, it remains to be determined whether MSCs derived from healthy or sarcoma affected-patients have functional defects that could hamper therapeutic efficacy. In this study, we evaluated the characteristics of BM-derived MSCs from sarcoma patients and healthy controls *in vitro* to assess their oncogenic potential before clinical application.

## Methods

### Study design

The *in vitro* biosafety profiles of BM-derived MSCs from OS and EWS patients (MSC-SAR) were compared to those of BM-MSCs from control healthy donors (MSC-CTRL) after expansion under the same culture conditions. Potential hallmarks of tumorigenic transformation were assessed by characterizing MSC morphology and immunophenotype, osteogenic and adipogenic differentiation, sequencing genes frequently mutated in OS and EWS, evaluating telomerase activity, assessing the gene expression profile of major cancer pathways, as well as cytogenetic analysis on synchronous MSCs, and molecular karyotyping using a comparative genomic hybridization (CGH) array.

### Patients

The study was approved by the Rizzoli Orthopedic Institute Ethics Committee (Bologna, Italy), and all patients provided informed consent. Seven bone sarcoma patients and six healthy donors were included. Detailed information about the bone sarcoma patients is shown in Table 1.

**Table 1:** Characteristics of patients and bone sarcomas

**Sample ID**	**Gender**	**Age**	**Diagnosis**	**Location**
MSC-SAR 1	F	36	Osteosarcoma	Proximal humerus
MSC-SAR 2	M	45	Osteosarcoma	Distal femour
MSC-SAR 3	M	17	Ewing Sarcoma	Iliac crest
MSC-SAR 4	M	20	Osteosarcoma	Distal femour
MSC-SAR 5	M	12	Ewing Sarcoma	Femour
MSC-SAR 6	M	63	Condrosarcoma	Acetabolar
MSC-SAR 7	M	17	Osteosarcoma	Femour

F, female; M, male.

### Isolation of bone marrow nucleated cells and MSCs expansion

Isolation of BM-derived MSCs was performed as described previously [29] through gradient separation and plastic adherence. Briefly, 8 mL of undiluted BM aspirate were loaded into a BD Vacutainer® CPT™ tube (Becton Dickinson, Franklin Lakes, NJ, USA) and then processed according to the manufacturer’s instructions. The top layer containing plasma and mononuclear cells was harvested. The cell number was counted and the viability evaluated. For expansion, cells were then transferred to 150-cm^2^ culture flasks by seeding 4 × 10^5^ cells/cm^2^ with α-Modified Minimum Essential medium (α-MEM; BioWhittaker, Lonza, Verviers, Belgium) supplemented with 20% lot-selected fetal bovine serum (FBS; Gibco, Invitrogen-Life Technologies, Paisley, UK) and 1% GlutaMAX™ (Invitrogen-Life Technologies). The flasks were incubated at 37°C in a humidified atmosphere of 5% CO_2_ with medium change every 3–4 days. When the cells reached ~70–80% confluence, they were detached by mild trypsinization (TripLe™ Select, Invitrogen-Life Technologies) for 3 min at 37°C and counted. Cells were reseeded into a new 150-cm^2^ flask at a density of 4000 cells/cm^2^.

### Immunophenotypic characterization

Phenotypic characterization of MSCs was performed by fluorescence-activated cell sorting (FACS) analysis of cell-surface markers at passage 2 (P2). MSCs were labeled with monoclonal antibodies against CD34, CD45, CD44, CD90, CD105, CD166 (Beckman Coulter, Fullerton, CA, USA) and CD146 (Miltenyi Biotech, Bergisch Gladbach, Germany). Control samples were labeled with isotype-matched control antibodies (Beckman Coulter, Brea, CA, USA). In brief, cells were trypsinized and aliquoted at a concentration of 1 × 10^6^ cells/mL, fixed in 0.5% formalin for 20 min, and washed once in PBS. Next, samples were incubated with either conjugated specific antibodies or isotype-matched control mouse immunoglobulin G at the recommended concentrations. Labeled cells were washed twice and suspended in FACS buffer. The analysis was performed using a FC500 flow cytometer (Beckman Coulter).

### Cell proliferation

Cell number and viability were assessed for each passage using a NucleoCounter® device (ChemoMetec, Lillerød, Denmark) that detects non-viable cells by propidium iodide nuclear staining and determines cell viability by calculating the ratio of non-viable to total cell numbers. The number of population doublings (PD) for each passage was calculated using the formula: *log*_*2*_*(N*_*1*_*/N*_*0*_*)*, where *N*_*0*_ is the number of cells seeded and *N*_*1*_ the number of cells harvested at the end of the passage and cumulative population doubling (CPD) refers to the sum of PDs over passages.

### Senescence assay

Senescence was detected by staining MSCs with a β-galactosidase (SA-β-gal) staining kit (Cell Signaling Technologies, Danvers, MA, USA) according to the manufacturer’s instructions, and analyzed with a direct-light microscope. Briefly, 1 × 10^4^ cells were plated in a 35-mm^2^ dish and incubated overnight at 37°C. After removing the growth medium, cells were washed twice with PBS, fixed for 10–15 min at room temperature, and incubated at 37°C overnight in a dry incubator (atmospheric CO_2_) with fresh β-gal staining solution. β-gal–positive cells were monitored under a microscope for the development of blue color and subsequently imaged.

### MSCs differentiation *in vitro*

Osteogenic differentiation was induced at P3 by seeding MSCs in α-MEM supplemented with 2% FBS in six-well plates at 5 × 10^5^ cells per well. The next day, an osteogenic-inducing cocktail composed of 10 mM β-glycerophosphate (Sigma, St. Louis, MO, USA), 50 μg/mL ascorbic acid (Sigma) and 100 nM dexamethasone (Sigma) was added. As a negative control, cells seeded under the same conditions were maintained in a non-inducing medium. Media were changed twice per week. After 14 days, the samples were stained with Alizarin Red-S (AR-S) (Sigma) to reveal the deposition of a calcium-rich mineralized matrix [30]. Adipogenic differentiation was induced at P3 by seeding 5 × 10^5^ MSCs/well in a six-well plate in Dulbecco’s Modified Essential Medium-high glucose (DMEM–HG; Euroclone, Milan, Italy) supplemented with 2% FBS (Gibco) and incubated overnight to allow cell attachment. Then, medium was switched to adipogenic-induction medium composed of DMEM–HG supplemented with 2% FBS, 10 μM bovine insulin (Sigma, St Louis, MO, USA), 1 μM dexamethasone (Sigma), 200 μM indomethacin (Sigma) and 500 μM 3-isobutyl-1-methyl xanthine (IBMX, Sigma). As a negative control, cells seeded under the same conditions were maintained in non-inducing medium. Media were changed twice per week. After 21 days, the presence of lipid depots was visualized by staining samples with Oil Red O. In brief, cells were washed twice with phosphate-buffered saline (PBS; Euroclone, Milan, Italy), fixed in 4% paraformaldehyde (Sigma) for 10 min and stained with 0.18% Oil Red O (Sigma) for 15 min.

### Cell-cycle synchronization and cytogenetic analyses

It has been reported [21] that 20–25 valuable metaphase cells/slides can be obtained by synchronizing MSCs. To synchronize the cell cycle, MSCs at > 80% confluence were detached, re-plated at 7000 cells/cm^2^, and maintained in culture medium without FBS for 20 h. After that, complete medium containing FBS was returned to the cultures for 27–28 h and incubated at 37°C with 0.1 mM colcemid solution (Irvine Scientific, Santa Ana, CA, USA). After 4 h, cells were harvested, treated with 0.56 mM KCl, and fixed in methanol/acetic acid (3:1). Cells in metaphase were Q-banded and karyotyped in accordance with the International System for Human Cytogenetic Nomenclature recommendations.

### CGH array

Molecular karyotyping was performed by CGH array with the Agilent kit 2 × 105 K (Human Genome CGH Microarray, v. 5.0, Agilent Technologies, Santa Clara, CA, USA), according to the manufacturer’s protocol, as described previously [24]. The analysis was performed on four MSC-SAR and four MSC-CTRL samples. The minimum positive criteria for an imbalance was considered to be three consecutive oligomeres with a log_2_ ratio different from zero; thus, the theoretical resolution of the 105 K 60-mer oligonucleotide platform was ~80 kb. DNA was extracted using QIAamp DNA Blood Mini Kit (Qiagen, Hilden, Germany) according to the manufacturer’s protocol. Array-CGH experiments were analyzed using the Agilent scanner and Feature Extraction software (v. 9.1). A graphical overview was obtained using the CGH Analytics software (v. 3.4.27). Quality control parameters for each experiment were evaluated using the QC metric tool in the CGH Analytics software.

### Telomerase activity assay

Telomerase activity was measured by a quantitative real-time PCR-based telomeric repeat amplification protocol (TRAP). MSC-SAR and MSC-CTRL were analyzed by the TeloExpress quantitative telomerase detection kit (Express Biotech International, Thurmont, USA), according to the manufacturer’s instructions. The Huh7 cell line was used as a telomerase-positive control. The results obtained from each MSC sample were compared with a control template standard curve for final absolute quantification of telomerase activity, expressed as attomole of telomerase repeat sequences in 1-μg protein (attomol/μg protein).

### Gene sequencing

MSCs obtained from cultures of sarcoma and healthy donors were harvested at early and late passages and washed in PBS buffer. DNA was isolated using the Nucleospin Blood kit (Macherey-Nagel, Dueren, Germany) according to manufacturer’s instructions. DNA quality and quantity were assessed with a NanoQuant Infinite M200 instrument (Tecan Group Ltd, Männedorf, Switzerland) before sequencing.

DNA samples were analyzed for mutational screening of *TP53*, *CDKN1A/p21* and *MDM2* genes. The 11 exons of *TP53*, the 3 exons of *CDKN1A* along with exon–intron junctions, and SNP309 (rs2279744) in *MDM2* were PCR-amplified using primer sequences that will be available upon request. The amplification products were purified using ExoSap-IT reagent (USB Corp., Cleveland, OH, USA) and sequenced in both the forward and reverse directions using BigDye Terminator chemistry version 3.1 (Applied Biosystems, Foster City, CA, USA). Purification of sequencing products was performed with BigDye X-Terminator kit and samples were analyzed using an ABI Prism 3100 automated DNA sequence (Applied Biosystems). Reference sequences for *TP53*, *CDKN1A*, and *MDM2* were obtained from GenBank (accession numbers NM_000546.4, NM_000389 and NM_002392.3, respectively).

### Gene expression analysis

Total RNA from MSC-SAR and MSC-CTRL harvested at early and late culture passages, as well as from subconfluent osteosarcoma cell line U2OS (#HTB-96, ATCC, Teddington, United Kingdom), was isolated using the Qiagen RNeasy mini kit (Qiagen) according to the manufacturer’s instructions. RNA concentration was assessed with a NanoQuant Infinite M200 instrument (Tecan Group Ltd, Männedorf, Switzerland) and RNA integrity was verified spectrophotometrically by 260/280 nm ratios > 2.0 and 260/230 nm ratios > 1.7. An equal amount of RNA (500 ng) was used for reverse transcription using the RT^2^ First Strand Kit (Qiagen) using protocol steps that eliminated genomic DNA.

qPCR experiments were performed using the Human Cancer Pathway Finder PCR Array (RT^2^ Profiler PCR Array PAHS-033R, SABioscience, Frederick, MD, USA) and RT^2^ SYBR Green ROX Fast Mastermix (Qiagen) on a Rotor-Gene Q instrument. The total volume of the PCR reaction was 20 μL and reactions were setup with a Biomek® NX Span-8 automated workstation (Beckman Coulter, Indianapolis, IN, USA) equipped with an adaptor designed to hold Rotor-disc 100 (Qiagen). The thermocycler parameters were 95°C for 10 min, followed by 40 cycles at 95°C for 15 s and then at 60°C for 30 s. The PCR array profiles the expression of 84 genes involved in transformation and tumorigenesis. Four housekeeping genes (*B2M, HPRT1, RPL13A, ACTB*), RT controls, and PCR controls were included in each run. Relative expression of target genes was determined using the ΔΔCq method, as described by Livak and Schmittgen [31]. PCR-array data were analyzed using the web-based software “RT2 Profiler PCR Array Data Analysis v. 3.5”, available at the manufacturer’s website [32]. The analysis was performed on four MSC-CTRL, four MSC-SAR, and three U2OS samples.

### Statistical analysis

Linear regression of CPD curves from MSC-SAR (n = 6) and MSC-CTRL (n = 6) was performed using GraphPad Prism version 5.00 for Windows (GraphPad Software, San Diego, California USA). The same software was used to compare the mean best-fit slope of two groups.

## Results

### Characterization of MSCs

**Figure 1:**
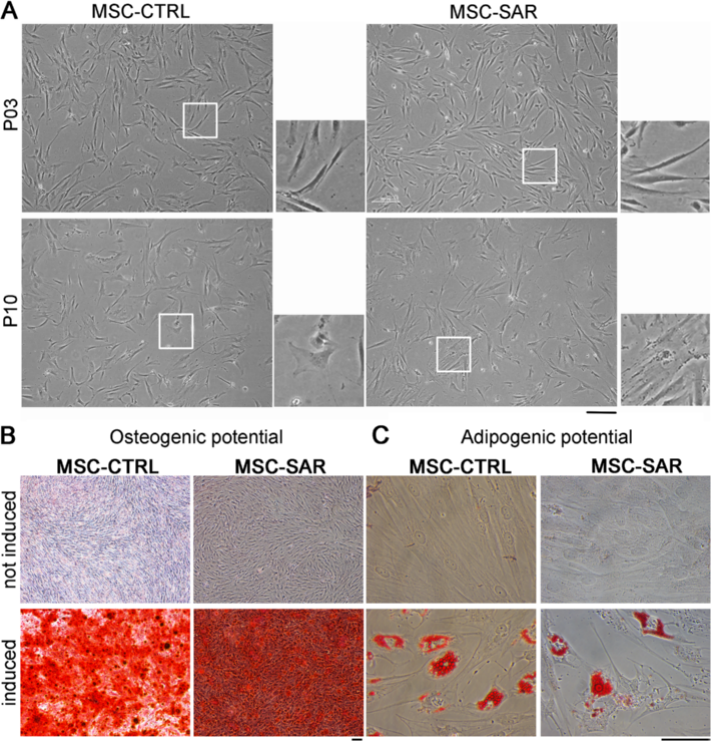
**Morphological analysis and *in vitro *differentiation of mesenchymal stem cells from control (MSC-CTRL) and sarcoma (MSC-SAR) patients. (A)** The morphology of MSC-CTRL and MSC-SAR samples with culture passage. Representative bright-field images of MSC-CTRL and MSC-SAR at early (P3) and late (P10) passages. White boxes indicate areas of magnification shown in the lateral panels. Scale bar = 100 μm. **(B)** Osteogenic differentiation assay of MSC-CTRL and MSC-SAR samples. Representative bright-field images of Alizarin Red staining of non-induced MSC-CTRL and MSC-SAR, and after 14 days in osteogenic medium. Red staining highlights deposition of mineralized extracellular matrix by differentiated MSCs. Scale bar = 100 μm. **(C)** Adipogenic differentiation assay of MSC-CTRL and MSC-SAR samples. Representative bright-field images of Oil Red O staining of non-induced MSC-CTRL and MSC-SAR and after 21-day culture in adipogenic medium. Red staining indicates fat deposits, a hallmark of fully differentiated adipocytes. Scale bar = 50 μm.

To characterize MSCs isolated from the BM of sarcoma patients and controls, we evaluated cell morphology, expression of typical surface markers and differentiation potential into mesodermal lineage cells. Both populations displayed the typical spindle-shaped appearance at early passages and exhibited a smoothened morphology after long-term expansion, developing a larger and more granular cytoplasm (Figure 1A). FACS analysis documented that > 80% of the MSC-CTRL and MSC-SAR expressed the typical MSC markers CD44, CD90, CD105, CD146, CD166, whereas the expression of the hematopoietic markers CD45 and CD34 was <10%. No quantitative differences were observed (data not shown). Furthermore, MSC-CTRL and MSC-SAR displayed the expected osteogenic (Figure 1B) and adipogenic differentiation potentials (Figure 1C).

### Growth kinetics are not altered in MSC-SAR

**Figure 2:**
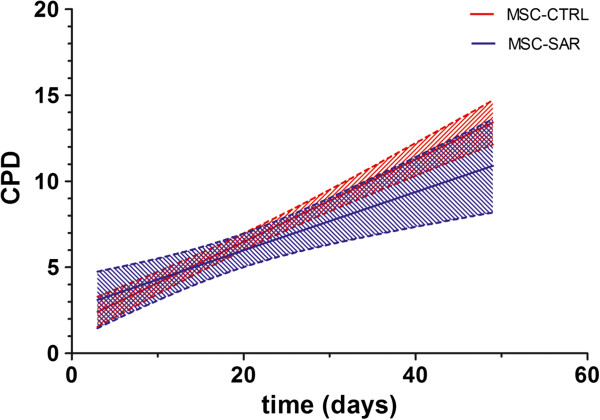
**Proliferation of mesenchymal stem cells from control (MSC-CTRL) and sarcoma (MSC-SAR) patients during *in vitro *expansion.** Cumulative population doubling (CPD) from MSC-CTRL (n = 6) and MSC-SAR (n = 6) during seven passages. Results are expressed as best-fit linear regression lines (solid lines) with 95% < confidence intervals (dashed lines).

**Figure 3:**
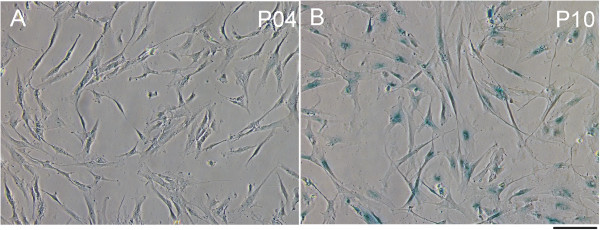
**Senescence assay.** Evaluation of β-galactosidase activity in MSC-SAR at passage **(A)** 4 (P4) and **(B)** 10 (P10). Senescent cells are stained blue. Scale bar = 100 μm.

We measured the proliferation rate of six MSC-CTRL and six MSC-SAR samples using standard growth curve analysis during 30 days in culture, which corresponded to seven passages. Despite the considerable variability in proliferation kinetics, cumulative population doubling (CPD) analysis revealed that the MSC-SAR proliferation rate from P3 to P10 was comparable to MSC-CTRL (Figure 2). Moreover, both MSC-CTRL and MSC-SAR, entered a senescent phase within 100 days (P7) of culture, as shown by β-gal staining (Figure 3). This was supported by the observation that a larger number of β-gal positive cells were observed at late (P10, Figure 3B) than at early (P4, Figure 3A) passages.

### MSC-SAR and MSC-CTRL exhibit chromosomal aberrations

**Figure 4:**
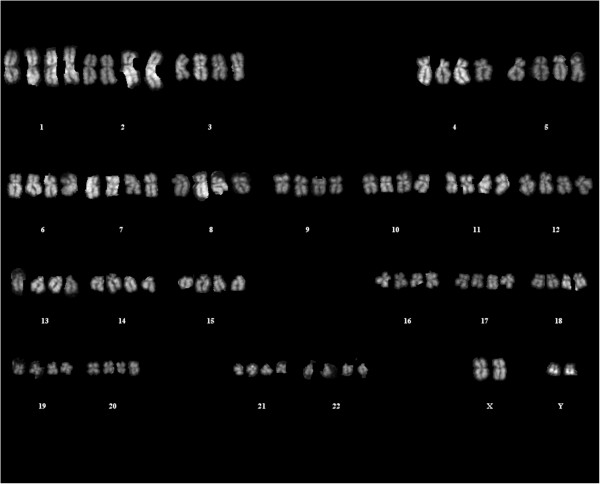
**Karyotype analysis.** Q-banding karyotype of representative MSC-SAR tetraploid cells analyzed in accordance with the International System for Human Cytogenetic Nomenclature recommendations.

The genetic stability of the *in vitro* expanded MSC-CTRL and MSC-SAR was evaluated after the *in vitro* expansion by both molecular and conventional karyotyping. CGH array analysis did not demonstrate any chromosomal abnormalities or sub-microscopic rearrangements (data not shown). However, CGH arrays cannot detect balanced chromosomal rearrangements, a problem which can be avoided by simultaneously performing a conventional cytogenetic analysis. In this case, conventional karyotyping did not detect macroscopic chromosomal rearrangement but did detect tetraploid cells in both early and late passages of MSC-SAR, as well as in MSC-CTRL cultures (Figure 4). Analysis of 211 metaphase cells obtained from five MSC-SAR lots detected 46 tetraploid cells (22%), while 8 tetraploid cells (18%) were detected within the 45 metaphase cells obtained from two MSC-CTRL lots. We could not identify chromosomal aberrations frequently associated with bone tissue tumors, such as monosomy 13 [33].

### MSC-SAR have low levels of telomerase activity

**Figure 5:**
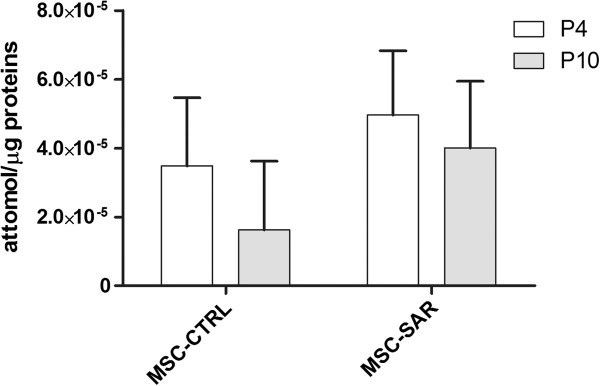
**Telomerase activity.** Telomerase activity of five MSC-SAR and five MSC-CTRL samples at P4 and P10, as detected by TRAP assay. Results are expressed as means ± standard deviation (n = 5).

To evaluate telomerase activity, we determined the ability of telomerase to synthesize telomeric repeats onto an oligonucleotide substrate *in vitro* upon the addition of deoxynucleotides (dNTPs). Extended products were subsequently amplified by PCR. Five MSC-CTRL and five MSC-SAR at early (P4) and late (P10) passages were tested. All MSC samples showed a passage-associated decrease of telomerase activity (Figure 5). The mean value in MSC-SAR was 4.96 × 10^-5^ ± 1.87 × 10^-5^ attomol/μg protein at P4 (range 3.6–8.5 × 10^-5^ attomol/μg protein) and 4 × 10^-5^ ± 1.94 × 10^-5^ attomol/μg protein at P10 (range 1.21–6.17 × 10^-5^ attomol/μg protein). The mean telomerase activity in MSC-CTRL cells was 3.48 × 10^-5^ ± 1.98 × 10^-5^ attomol/μg protein at P4 (range 3–7.2 × 10^-5^ attomol/μg protein) and 1.62 × 10^-5^ ± 2 × 10^-5^ attomol/μg protein at P10 (range 0.9–3.04 × 10^-5^ attomol/μg protein).

### MSC-SAR do not have specific DNA sequence alterations

Direct sequencing of all exonic and intronic boundaries of *TP53* and *CDKN1A*, along with *MDM2* SNP309, was performed in order to evaluate sequence variations in DNA samples from MSC-CTRL and MSC-SAR harvested at early and late passages (see Table 2 for details). Deficiencies in *TP53* and *CDKN1A*, the primary regulators of cell cycle progression and apoptosis normally involved in protection against tumorigenesis, can be the origin of some mesodermic tumors [34,35]. Since *MDM2* encodes an important negative regulator of the p53 protein, we also assessed the status of *MDM2* SNP309 (rs2279744), which is located in the *MDM2* promoter. All donors analyzed showed the presence of exonic and/or intronic polymorphisms in *TP53* (Table 2). All of these variants were reported in the *TP53* database as benign sequence variations [36]. *CDKN1A* sequencing revealed only one sequence variant in MSC-SAR 4 (rs1059234), which had been described previously [37], and is not associated with cancer [38]. The heterozygous presence of SNP309 (rs2279744 T > G) in *MDM2* was detected in three donors (Table 2). No pathological mutations were found in the analyzed genes. Notably, all *TP53*, *CDKN1A*, and *MDM2* variants identified in each donor were detected in all DNA samples analyzed. Moreover, the hetero- or homozygous status of these variations did not change at different culture time points. In addition, to detect the presence of the EWS/FLI-1 fusion transcript, we performed RT-PCR amplification using primers spanning the EWS/FLI-1 fusion region [39] on MSC-SAR derived from EWS-affected patients (MSC-SAR 3 and 5). Interestingly, we did not detect EWS/FLI-1 expression in either sample (data not shown).

**Table 2:** ***p53*, *CDKN1A *** **and *MDM2 *** **sequence analysis**

**Donor ID (passages analyzed)**	**TP53**			**CDKN1A p21**			**MDM2 SNP 309**	
	**refSNP**	**Exon**	**Status**	**refSNP**	**Exon**	**Status**	**refSNP**	**Status**
**MSC-SAR 1 (p4/p10)**	rs1642785	2	HO					
	rs17878362	3	HO					
	rs1042522	4	HO					
**MSC-SAR 2 (p4/p10)**	rs1642785	2	HE				rs2279745	HE
	rs17878362	3	HO					
	rs1042522	4	HE					
**MSC-SAR 3 (p4/p6)**	rs1642785	2	HO					
	rs17878362	3	HO					
	rs1042522	4	HO					
**MSC-SAR 4 (p4/p10)**	rs1642785	2	HE	rs1059234	3	HE		
	rs17878362	3	HE					
	rs1800370	4	HE					
	rs1042522	4	HE					
**MSC-SAR 5 (p4/p10)**	rs1642785	2	HE				rs2279745	HE
	rs17878362	3	HO					
	rs1042522	4	HE					
	rs12947788	7	HE					
	rs12951053	7	HE					
**MSC-SAR 6 (p4/p10)**	rs17878362	3	HO				rs2279745	HE
	rs12947788	7	HE					
	rs12951053	7	HE					
**MSC-CTRL 1 (p4/p10)**	rs1642785	2	HO					
	rs17878362	3	HO					
	rs1042522	4	HO					
**MSC-CTRL 2 (p3/p10)**	rs1642785	2	HO					
	rs17878362	3	HO					
	rs1042522	4	HO					

Sequence analysis of DNA samples from MSC-SAR and MSC-CTRL patients was performed at the indicated culture passages. PCR amplification was performed on the 11 exons of *TP53*, the 3 exons of *CDKN1A* along with exon–intron junctions, and on the SNP309 (rs2279744) in *MDM2*. P, passages; HO, homozygous; HE, heterozygous.

### Gene expression in cancer pathways was not significantly altered in MSC-SAR

**Figure 6:**
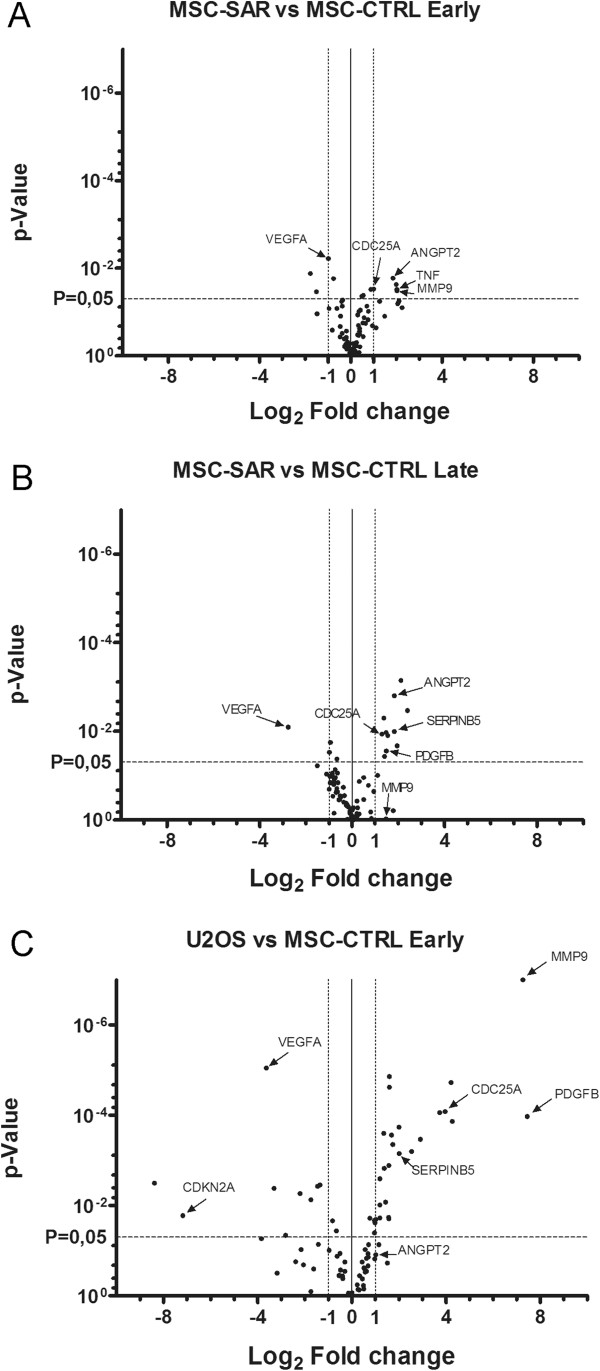
**Gene expression analysis of cancer pathways.** Gene expression analysis was performed using the RT^2^ Profiler PCR Array PAHS-033R SABioscience. Volcano plots of MSC-SAR samples (n = 4) compared to MSC-CTRL (n = 4) at early **(A)** and late **(B)** passages are reported. The volcano plot of the U2OS tumor cells versus MSC-CTRL samples is shown in **(C)**. Also see Table 3.

To identify a possible variation in markers correlated previously to tumorigenesis and determine whether these markers were specifically associated to bone sarcoma etiology, we investigated gene expression levels in MSC-CTRL and MSC-SAR samples at different passages after the *in vitro* culture (see Table 3 for details). The analysis was performed using the RT^2^ Profiler PCR Array by SABioscience, which is designed to characterize 84 genes representative of biological pathways involved in transformation and tumorigenesis, cell cycle control and DNA damage repair, apoptosis and cell senescence, signal transduction molecules and transcription factors, adhesion, angiogenesis, invasion and metastasis. Results from this analysis are shown in table form (Additional file 1: Table S1) and graphically (Figure 6). Altered gene expression was defined as up- or down-regulation ≥ 2-fold and a p value ≤ 0.05. The expression of 15 genes (7 genes at P3 and 11 at P10) was significantly different in MSC-SAR than in MSC-CTRL. Over- or under-expression of these genes are presented as fold regulation in Table 3, along with corresponding results obtained by comparing osteosarcoma cells from the reference tumor cell line U2OS with MSC-CTRL. At P3, *MMP9* and *TNF* were overexpressed genes in MSC-SAR (Figure 6A and Table 3). However, at late passages no differences in gene expression were observed. We did observe that increased expression of *ANGPT2* and C*DC25A* was maintained throughout culture passages (Figure 6A,B); however, these genes are not correlated with bone sarcoma etiology.

**Table 3:** Gene expression analysis of cancer pathways

			**MSC-SAR vs MSC-CTRL P03**		**MSC-SAR vs MSC-CTRL P10**		**U2OS vs MSC-CTRL P03**	
**GENE SYMBOL**	**Refseq**	**Description**	**Fold Regulation**	**T-TEST p value**	**Fold Regulation**	**T-TEST p value**	**Fold Regulation**	**T-TEST p value**
**ANGPT2**	NM_001147	Angiopoietin 2	**3,60**	**0,017031**	**3,54**	**0,001587**	2,01	0,123058
**BRCA1**	NM_007294	Breast cancer 1, early onset	1,56	0,184634	**2,59**	**0,005080**	**13,19**	**0,000088**
**CDC25A**	NM_001789	Cell division cycle 25 homolog A (S. pombe)	**2,01**	**0,030181**	**2,44**	**0,011524**	**15,56**	**0,000083**
**CFLAR**	NM_003879	CASP8 and FADD-like apoptosis regulator	**-3,43**	**0,013343**	-1,91	0,017928	-1,42	0,115505
**CHEK2**	NM_007194	CHK2 checkpoint homolog (S. pombe)	1,03	0,930306	1,23	0,735103	**7,49**	**0,000340**
**COL18A1**	NM_030582	Collagen, type XVIII, alpha 1	**-2,85**	**0,034861**	-1,14	0,967097	**2,94**	**0,018415**
**E2F1**	NM_005225	E2F transcription factor 1	1,69	0,152813	**2,64**	**0,036988**	**18,50**	**0,000019**
**GZMA**	NM_006144	Granzyme A (granzyme 1, cytotoxic T-lymphocyte-associated serine esterase 3)	4,14	0,064832	**3,86**	**0,021397**	**2,28**	**0,018937**
**IFNA1**	NM_024013	Interferon, alpha 1	2,15	0,231676	**4,32**	**0,000719**	-4,17	0,208829
**IGF1**	NM_000618	Insulin-like growth factor 1 (somatomedin C)	**3,98**	**0,023798**	**5,26**	**0,003415**	1,41	0,704213
**MMP9**	NM_004994	Matrix metallopeptidase 9 (gelatinase B, 92 kDa gelatinase, 92 kDa type IV collagenase)	**4,05**	**0,033049**	2,76	0,957572	**152,53**	**0,000000**
**PDGFB**	NM_002608	Platelet-derived growth factor beta polypeptide	2,41	0,057864	**2,80**	**0,027891**	**174,40**	**0,000107**
**SERPINB5**	NM_002639	Serpin peptidase inhibitor, clade B (ovalbumin), member 5	4,32	0,056954	**3,54**	**0,010117**	**4,04**	**0,000712**
**TNF**	NM_000594	Tumor necrosis factor	**4,04**	**0,030749**	**2,77**	**0,010705**	1,54	0,285973
**VEGFA**	NM_003376	Vascular endothelial growth factor A	-1,97	0,006089	**-6,84**	**0,008108**	**-12,44**	**0,000009**

Genes differentially expressed in MSC-SAR compared to MSC-CTRL at early and late passages characterized in the Human Cancer Pathway Finder PCR Array (RT^2^ Profiler PCR Array PAHS-033R, SABioscience), and analyzed using the provided RT^2^ Profiler PCR Array Data Analysis v. 3.5 software [32]. Results of the same genes analyzed in U2OS cells are reported for comparison. Bold characters indicate genes up- or down-regulated by ≥ 2-fold and with a p-value ≤ 0.05. The analysis was performed on four MSC-CTRL, four MSC-SAR samples and three independent samples of sub-confluent U2OS cells.

Our analysis indicated that expression of platelet-derived growth factor B (*PDFGB*), which is involved in the regulation of proliferation in cells of mesenchymal origin, increases over time during culture in MSC-SAR (Figure 6B). Moreover, expression of the tumor suppressor gene *SERPINB5* was higher in MSC-SAR than in MSC-CTRL at late culture passages (Figure 6B). Conversely, vascular endothelial growth factor A (*VEGFA*) gene was slightly underexpressed in MSC-SAR at early stages of culture (Figure 6A) and decreased with time in culture (Figure 6B).

As expected when we compared U2OS with MSC-CTRL, we found a higher number of genes with greater levels of dysregulation. Of the 84 genes evaluated, we detected 33 that were altered (Figure 6C, Additional file 1: Table S1). U2OS cells strongly overexpressed *MMP9* (152-fold increase), confirming the high metastatic potential of this cell line coupled with high expression levels of genes involved in cell cycle regulation (*i.e., BRCA1, CCNE1, CDC25A, CHEK2, E2F1*), apoptosis (*i.e., BCL2, BCL2L1*), membrane integrity and tumor invasion (*i.e., COL18A1, ITGA3, MMP9, MCAM*), cell proliferation (*i.e.,. PDFGA, PDGFB*), and tumor suppression (*i.e., SERPINB5*). Among the underexpressed genes, p16 (*CDKN2A*) exhibited the greatest downregulation (145-fold decrease), which confirmed that the loss of p16 expression is correlated with osteosarcoma development [40]. Moreover, we confirmed reports in the literature describing over-expression of *PDGFB *[41].

## Discussion

The most common surgical techniques used to treat osteosarcoma around the knee include resection of the epiphysis and reconstruction with a prosthesis, an osteoarticular allograft, an autoclaved autograft, or a combination of the above. The functional results of prostheses and osteoarticular grafts are not satisfactory because of limited durability, joint instability, incongruity, and gross distortion of the normal anatomy. It has been reported that autologous MSCs loaded onto hydroxyapatite scaffolds can successfully heal segmental bone defects in human and animal models [42-44]. In human bone diseases, MSCs are usually delivered or applied locally, often in combination with suitable scaffolds, when it is necessary to provide mechanical stabilization or support to osteosynthesized fractures of long bones [45] and in atrophic non-unions. Although controversial, MSCs seeded on hydroxyapatite scaffolds have also been used to heal defects derived from curettage of a bone tumor as an alternative to autologous bone grafting [46]. However, the possible risks of MCSs transplantation are debated. Major concerns have been raised with regard to the biosafety of the *in vitro* expanded MSCs, particularly when intended for autologous transplantation in a cell therapy protocol for bone reconstruction of sarcoma patients. Data from a mouse xenograft model proposed that MSCs are precursors of tumor stromal cells [47] or might differentiate into tumor-associated fibroblast-like cells when cultured in tumor cell-conditioned supernatant [48]. Therefore, our *in vitro* studies will facilitate the safe use of expanded autologous MSCs for tissue engineering strategies to induce bone reformation.

In the present work, we performed cell expansion experiments under previously standardized culture conditions [29] and assessed *in vitro* the biosafety profile of MSCs isolated from the BM of sarcoma patients compared to control donors. Based on the “hallmarks of cancer” criterion [49], human cells acquire biological capabilities during tumor development in a multi-step process. These hallmarks include sustaining proliferative signaling, evading growth suppressors and enabling replicative immortality, which are all traits found in OS cell lines. We investigated the hallmarks of cancer in MSC-SAR at several levels; the results suggest that MSC-SAR exhibit comparable morphology, immunophenotype, proliferation rate, differentiation potential, and telomerase activity to MSCs of healthy donors. The *in vitro* expansion of both MSC-SAR and MSC-CTRL resulted in a progressive aging mechanism coupled to typical traits of altered cell morphology that are consistent with a previous study [50].

DNA sequencing of *TP53*, *CDKN1A*/p21 and *MDM2*, which are key players in cell cycle regulation and are involved in tumor of mesenchymal origin, did not detect pathological mutations. In addition, the existence of tetraploid cells in both early and late passages of MSCs cultures raises the crucial question whether MSCs clones with a genomic imbalance may acquire a malignant phenotype *in vivo*, although they are able to reach the senescence phase *in vitro*. The detection of tetraploid cells at similar percentages from MSC-SAR and MSC-CTRL suggests that this chromosomal aberration is not a distinctive feature of MSCs expanded *in vitro* from the BM of sarcoma patients. Rather, this chromosomal aberration may be induced by *in vitro* culture conditions during expansion procedures that are optimized to achieve a high proliferation rate and to obtain the large number of cells necessary for the analyses and for cell-based therapy approaches. Moreover, it is worth considering that polyploidies were already present in early culture passages, but these positive clones did not acquire a proliferative advantage during culture. These data are in agreement with a previous report by Tarte *et al. *[51] documenting, by conventional karyotype analysis, donor-dependent chromosomal abnormalities in healthy donor BM-MSCs that did not confer a selective advantage to the affected clone.

Gene expression analysis of 84 genes involved in cancer development provided a comparison of MSC-SAR and MSC-CTRL at a translational level. In this study, we used U2OS as a reference tumor cell line and, as expected, 33 of 84 genes investigated were altered when compared to MSC-CTRL. Interestingly, we observed down-regulation of p16 expression, which is responsible for escape from senescence and restoration of cell proliferation activity. Furthermore, several genes were overexpressed > 100-fold in U2OS compared to MSC-CTRL. As an example, MMP9 was overexpressed 152-fold, which is pertinent since MMPs are involved primarily in the breakdown of extracellular matrix and possibly promotion of tumor invasion.

A comparison of gene expression between MSC-SAR and MSC-CTRL revealed that the expression of 15 genes was significantly different, although none of these genes are principally involved in bone sarcoma etiology. While the expression of these 15 genes was altered in U2OS compared to MSC-CTRL, there was no difference between MSC-SAR and MSC-CTRL. For example, *MMP9* was increased 4.05-fold in MSC-SAR compared to MSC-CTRL at P3, even though no significant difference in *MMP9* expression was found at P10, suggesting that an increase in *MMP9* expression in MSC-SAR was not a stable indicator. *ANGPT2* and *CDC25A* maintain higher expression levels during *in vitro* culture in MSC-SAR compared to MSC-CTRL, but expression of these genes has not been correlated with bone sarcoma etiology (Figure 6A,B). Our analysis determined that expression of the mitogenic growth factor PDFGB increased with time in culture in MSC-SAR (Figure 6B). Moreover, the level of the tumor suppressor gene *SERPINB5* was higher in MSC-SAR at late culture passages (Figure 6B). Conversely, *VEGFA* was slightly underexpressed in MSC-SAR at early stages of culture (Figure 6A), and decreased over time (Figure 6B).

Gene expression analysis could provide a signature that will facilitate routine evaluation of the safety of *in vitro-*expanded MSCs and assessment of the presence of suspicious modifications. Our results support the hypothesis that MSC-SAR do not present a greater risk of undergoing transformation compared to MSC-CTRL. More extensive analysis should be performed to confirm the dysregulation of cancer pathways and exclude possible effects resulting from MSCs aging. The expression of tumor suppressor genes and oncogenes may in fact shift with time in culture and be influenced by stress response mechanisms that are activated under *in vitro* culture conditions. Our encouraging *in vitro* results will be expanded upon using *in vivo* approaches to confirm MSC-SAR safety in an animal model through long-term follow-up and careful examination of the transplanted animals.

## Conclusions

In conclusion, our findings suggest that BM-MSC derived from OS or EWS patients that are expanded *in vitro* are not prone to malignant transformation during culture. Nevertheless, the considerable percentage of cells displaying a chromosomal abnormality strongly indicates the necessity for genomic monitoring and rigorous evaluation of the biosafety profiles of the lots prepared for cell therapy. Consequently, the risks must be weighed carefully against the potential benefits to the patient.

## Abbreviations

BM: Bone marrow; EWS: Ewing sarcoma; MSCs: Mesenchymal stem cells; MSC-SAR: MSCs from BM sarcoma patients; MSC-CTRL: MSCs from BM healthy patients; OS: Osteosarcoma.

## Competing interests

The authors declare that they have no competing interests.

## Authors’ contributions

EL, SD and CB carried out the isolation and *in vitro* expansion of MSC-CTRL and MSC-SAR, performed immunophenotypic characterization, cell proliferation analysis, and MSC differentiation *in vitro.* SD performed gene expression analysis, while CB analyzed the data and performed the statistical analysis. SD drafted the manuscript and EL coordinated the study and assisted in drafting the manuscript. MM, MAA and RM participated in the *in vitro* expansion of MSC-SAR and MSC-CTRL to perform the senescence assay and cytogenetic analyses of cell-cycle synchronized cultures. FN, GA and OZ *in vitro* expanded the MSC cultures and performed CGH array and the corresponding data interpretation. MZ, MP and LS carried out the DNA sequencing analyses and the relative data interpretation. DL performed the telomerase activity assay. DD carried out the BM harvest of all collected MSC samples (MSC-SAR and MSC-CTRL) used in this paper, conceived the study and participated in its design. All authors read and approved the final manuscript.

## Supplementary Material

Additional file 1: Table S1 Gene expression analysis of cancer pathways. The 84 genes characterized in the Human Cancer Pathway Finder PCR Array (RT^2^ Profiler PCR Array PAHS-033R, SABioscience), and analyzed using the provided RT^2^ Profiler PCR Array Data Analysis v. 3.5 software [32]. Bold characters indicate genes up- or down-regulated by ≥ 2-fold and with a p-value ≤ 0.05. The analysis was performed on four MSC-CTRL, four MSC-SAR samples and three independent samples of sub-confluent U2OS cells. Click here for file
