# Supplementary material for: In vitro biosafety profile evaluation of multipotent mesenchymal stem cells derived from the bone marrow of sarcoma patients
Source: J Transl Med. 2014 Apr 9;12:95. doi: 10.1186/1479-5876-12-95 (PMC4022272; doi:10.1186/1479-5876-12-95)
Supplement: Additional file 1: Table S1 — Gene expression analysis of cancer pathways. The 84 genes characterized in the Human Cancer Pathway Finder PCR Array (RT2 Profiler PCR Array PAHS-033R, SABioscience), and analyzed using the provided RT2 Profiler PCR Array Data Analysis v. 3.5 software [32]. Bold characters indicate genes up- or down-regulated by ≥ 2-fold and with a p-value ≤ 0.05. The analysis was performed on four MSC-CTRL, four MSC-SAR samples and three independent samples of sub-confluent U2OS cells. [file 1479-5876-12-95-S1.pdf]

| | MSC-SAR early | MSC-SAR late | MSC-CTRL early | MSC-CTRL late | U2OS | MSC-SAR vs MSC-CTRL early | | MSC-SAR vs MSC-CTRL late | | U2OS vs MSC-CTRL early | |
|---|---|---|---|---|---|---|---|---|---|---|---|
| **GENE SYMBOL** | Average 2^-$\Delta C_t$ | Average 2^-$\Delta C_t$ | Average 2^-$\Delta C_t$ | Average 2^-$\Delta C_t$ | Average 2^-$\Delta C_t$ | Fold Regulation | T-TEST p value | Fold Regulation | T-TEST p value | Fold Regulation | T-TEST p value |
| **AKT1** | 6,97E-02 | 4,72E-02 | 7,13E-02 | 8,53E-02 | 5,41E-02 | -1,02 | 0,765104 | -1,81 | 0,088625 | -1,32 | 0,364970 |
| **ANGPT1** | 4,32E-03 | 4,53E-03 | 3,98E-03 | 3,21E-03 | 1,18E-03 | 1,08 | 0,677916 | 1,41 | 0,348774 | -3,37 | 0,809173 |
| **ANGPT2** | 9,51E-04 | 8,95E-04 | 2,65E-04 | 2,53E-04 | 5,33E-04 | **3,60** | **0,017031** | **3,54** | **0,001587** | 2,01 | 0,123058 |
| **APAF1** | 1,15E-02 | 8,09E-03 | 1,04E-02 | 7,00E-03 | 1,55E-02 | 1,10 | 0,975306 | 1,16 | 0,375459 | 1,48 | 0,269688 |
| **ATM** | 8,52E-03 | 6,23E-03 | 6,54E-03 | 5,86E-03 | 1,05E-02 | 1,30 | 0,325242 | 1,06 | 0,988743 | 1,61 | 0,116892 |
| **BAD** | 3,75E-02 | 3,36E-02 | 4,90E-02 | 5,31E-02 | 4,95E-02 | -1,30 | 0,312895 | -1,58 | 0,195335 | 1,01 | 0,999798 |
| **BAX** | 1,32E-01 | 1,08E-01 | 1,43E-01 | 1,30E-01 | 2,19E-01 | -1,08 | 0,594184 | -1,20 | 0,415719 | 1,53 | 0,149468 |
| **BCL2** | 2,41E-03 | 1,13E-03 | 1,41E-03 | 6,30E-04 | 8,12E-03 | 1,71 | 0,097942 | 1,79 | 0,929205 | **5,77** | **0,000639** |
| **BCL2L1** | 1,10E-02 | 1,28E-02 | 1,27E-02 | 2,21E-02 | 3,77E-02 | -1,15 | 0,387182 | -1,73 | 0,146070 | **2,96** | **0,019536** |
| **BRCA1** | 5,16E-03 | 4,29E-03 | 3,30E-03 | 1,66E-03 | 4,35E-02 | 1,56 | 0,184634 | **2,59** | **0,005080** | **13,19** | **0,000088** |
| **CASP8** | 3,21E-03 | 2,57E-03 | 2,44E-03 | 1,83E-03 | 4,75E-03 | 1,31 | 0,628797 | 1,41 | 0,111156 | 1,95 | 0,152393 |
| **CCNE1** | 1,43E-02 | 1,19E-02 | 1,02E-02 | 1,21E-02 | 1,96E-01 | 1,39 | 0,044333 | -1,02 | 0,872607 | **19,11** | **0,000138** |
| **CDC25A** | 3,96E-03 | 3,68E-03 | 1,97E-03 | 1,51E-03 | 3,06E-02 | **2,01** | **0,030181** | **2,44** | **0,011524** | **15,56** | **0,000083** |
| **CDK2** | 2,42E-02 | 1,93E-02 | 2,24E-02 | 1,75E-02 | 8,93E-02 | 1,08 | 0,532852 | 1,10 | 0,832247 | **3,99** | **0,000184** |
| **CDK4** | 1,26E-01 | 1,17E-01 | 1,61E-01 | 1,24E-01 | 4,11E-01 | -1,28 | 0,053576 | -1,06 | 0,612739 | **2,55** | **0,000252** |
| **CDKN1A** | 5,47E-01 | 5,70E-01 | 8,38E-01 | 9,48E-01 | 1,81E-01 | -1,53 | 0,083850 | -1,66 | 0,073162 | **-4,64** | **0,005422** |
| **CDKN2A** | 2,18E-02 | 2,44E-02 | 2,18E-02 | 4,17E-02 | 1,50E-04 | 1,00 | 0,978614 | -1,71 | 0,120455 | **-145,22** | **0,016905** |
| **CFLAR** | 1,12E-02 | 2,63E-02 | 3,85E-02 | 5,02E-02 | 2,70E-02 | **-3,43** | **0,013343** | -1,91 | 0,017928 | -1,42 | 0,115505 |
| **CHEK2** | 5,07E-03 | 4,64E-03 | 4,92E-03 | 3,78E-03 | 3,69E-02 | 1,03 | 0,930306 | 1,23 | 0,735103 | **7,49** | **0,000340** |
| **COL18A1** | 4,54E-03 | 4,18E-03 | 1,29E-02 | 4,78E-03 | 3,80E-02 | **-2,85** | **0,034861** | -1,14 | 0,967097 | **2,94** | **0,018415** |
| **E2F1** | 6,49E-03 | 4,76E-03 | 3,85E-03 | 1,81E-03 | 7,12E-02 | 1,69 | 0,152813 | **2,64** | **0,036988** | **18,50** | **0,000019** |
| **ERBB2** | 2,22E-02 | 1,57E-02 | 2,25E-02 | 2,53E-02 | 5,81E-02 | -1,01 | 0,902928 | -1,61 | 0,241704 | **2,59** | **0,001500** |
| **ETS2** | 4,53E-03 | 3,70E-03 | 5,09E-03 | 4,81E-03 | 1,53E-02 | -1,12 | 0,710765 | -1,30 | 0,183752 | **3,01** | **0,000024** |
| **FAS** | 3,82E-02 | 4,26E-02 | 2,99E-02 | 2,43E-02 | 9,72E-03 | 1,28 | 0,876118 | 1,75 | 0,664026 | -3,08 | 0,256075 |
| **FGFR2** | 5,88E-02 | 2,27E-02 | 5,85E-02 | 6,61E-03 | 4,00E-02 | 1,01 | 0,741693 | 3,44 | 0,620477 | -1,46 | 0,360448 |
| **FOS** | 2,07E-01 | 4,36E-02 | 2,10E-01 | 6,48E-02 | 2,59E-01 | -1,01 | 0,775771 | -1,48 | 0,351475 | 1,23 | 0,750323 |
| **GZMA** | 9,35E-04 | 8,60E-04 | 2,26E-04 | 2,23E-04 | 5,14E-04 | 4,14 | 0,064832 | **3,86** | **0,021397** | **2,28** | **0,018937** |
| **HTATIP2** | 2,15E-02 | 1,84E-02 | 2,39E-02 | 2,64E-02 | 4,65E-02 | -1,11 | 0,490396 | -1,43 | 0,287392 | 1,94 | 0,040669 |
| **IFNA1** | 2,20E-03 | 3,05E-03 | 1,02E-03 | 7,06E-04 | 2,45E-04 | 2,15 | 0,231676 | **4,32** | **0,000719** | -4,17 | 0,208829 |
| **IFNB1** | 4,63E-04 | 4,70E-04 | 1,66E-04 | 2,18E-04 | 2,45E-04 | 2,79 | 0,127232 | 2,16 | 0,099512 | 1,48 | 0,298755 |
| **IGF1** | 1,49E-03 | 1,01E-03 | 3,74E-04 | 1,92E-04 | 5,29E-04 | **3,98** | **0,023798** | **5,26** | **0,003415** | 1,41 | 0,704213 |
| **IL8** | 4,17E-03 | 4,22E-03 | 7,33E-03 | 8,38E-03 | 8,72E-03 | -1,76 | 0,263177 | -1,99 | 0,204722 | 1,19 | 0,725585 |
| **ITGA1** | 1,73E-02 | 1,02E-02 | 2,12E-02 | 1,01E-02 | 4,74E-03 | -1,22 | 0,412980 | 1,01 | 0,594644 | -4,47 | 0,094827 |
| **ITGA2** | 8,73E-03 | 1,38E-02 | 1,00E-02 | 1,36E-02 | 2,85E-02 | -1,15 | 0,382904 | 1,02 | 0,854478 | 2,84 | 0,189794 |
| **ITGA3** | 1,04E-01 | 1,09E-01 | 1,22E-01 | 1,66E-01 | 4,07E-01 | -1,17 | 0,572579 | -1,53 | 0,144098 | **3,33** | **0,000448** |
| **ITGA4** | 9,48E-03 | 1,08E-02 | 7,99E-03 | 1,11E-02 | 5,69E-03 | 1,19 | 0,593399 | -1,03 | 0,779080 | -1,40 | 0,271405 |
| **ITGAV** | 3,88E-01 | 2,64E-01 | 4,02E-01 | 4,17E-01 | 1,20E-01 | -1,03 | 0,870638 | -1,58 | 0,042175 | **-3,35** | **0,007472** |
| **ITGB1** | 3,04E+00 | 2,15E+00 | 3,30E+00 | 3,94E+00 | 2,67E+00 | -1,09 | 0,515971 | -1,83 | 0,104305 | -1,23 | 0,287381 |
| **ITGB3** | 2,01E-02 | 6,97E-03 | 1,62E-02 | 7,44E-03 | 9,16E-03 | 1,24 | 0,117479 | -1,07 | 0,633702 | -1,77 | 0,021870 |
| **ITGB5** | 2,93E-01 | 2,56E-01 | 4,01E-01 | 4,68E-01 | 4,03E-01 | -1,37 | 0,125478 | -1,83 | 0,089895 | 1,00 | 0,863627 |
| **JUN** | 2,16E-01 | 9,70E-02 | 2,16E-01 | 1,85E-01 | 2,92E-01 | 1,00 | 0,726263 | -1,91 | 0,143706 | 1,35 | 0,420094 |
| **MAP2K1** | 1,14E-01 | 1,14E-01 | 1,51E-01 | 2,25E-01 | 1,22E-01 | -1,32 | 0,058341 | -1,97 | 0,029927 | -1,24 | 0,178784 |
| **MCAM** | 1,76E-01 | 7,34E-02 | 1,21E-01 | 1,14E-01 | 2,75E-01 | 1,46 | 0,042330 | -1,56 | 0,231569 | **2,28** | **0,002570** |

| | MSC-SAR early | MSC-SAR late | MSC-CTRL early | MSC-CTRL late | U2OS | MSC-SAR vs MSC-CTRL early | | MSC-SAR vs MSC-CTRL late | | U2OS vs MSC-CTRL early | |
|---|---|---|---|---|---|---|---|---|---|---|---|
| **GENE SYMBOL** | Average $2^{-\Delta C_t}$ | Average $2^{-\Delta C_t}$ | Average $2^{-\Delta C_t}$ | Average $2^{-\Delta C_t}$ | Average $2^{-\Delta C_t}$ | Fold Regulation | T-TEST p value | Fold Regulation | T-TEST p value | Fold Regulation | T-TEST p value |
| **MDM2** | 7,73E-02 | 8,89E-02 | 6,81E-02 | 8,94E-02 | 1,38E-01 | 1,14 | 0,841048 | -1,01 | 0,977805 | **2,02** | **0,020313** |
| **MET** | 2,38E-01 | 2,38E-01 | 2,11E-01 | 2,76E-01 | 1,07E-01 | 1,13 | 0,887770 | -1,16 | 0,452837 | -1,97 | 0,098266 |
| **MMP1** | 1,88E-03 | 1,59E-03 | 2,16E-03 | 2,83E-03 | 2,39E-04 | -1,15 | 0,276776 | -1,78 | 0,294643 | -9,06 | 0,316661 |
| **MMP2** | 1,90E+00 | 1,11E+00 | 1,49E+00 | 1,18E+00 | 5,40E-01 | 1,28 | 0,098427 | -1,06 | 0,709936 | **-2,76** | **0,003769** |
| **MMP9** | 6,79E-04 | 7,60E-04 | 1,67E-04 | 2,75E-04 | 2,55E-02 | **4,05** | **0,033049** | 2,76 | 0,957572 | **152,53** | **0,000000** |
| **MTA1** | 7,99E-02 | 6,04E-02 | 6,15E-02 | 6,45E-02 | 1,00E-01 | 1,30 | 0,282030 | -1,07 | 0,672353 | 1,63 | 0,073407 |
| **MTA2** | 2,65E-02 | 1,96E-02 | 1,85E-02 | 2,30E-02 | 5,92E-02 | 1,44 | 0,180078 | -1,17 | 0,482202 | **3,21** | **0,000275** |
| **MTSS1** | 3,10E-03 | 3,75E-03 | 6,02E-03 | 6,47E-03 | 2,23E-03 | -1,94 | 0,084199 | -1,73 | 0,159863 | -2,70 | 0,073110 |
| **MYC** | 3,08E-02 | 2,08E-02 | 3,01E-02 | 3,00E-02 | 4,27E-02 | 1,02 | 0,945403 | -1,44 | 0,348538 | 1,42 | 0,244606 |
| **NFKB1** | 3,24E-02 | 3,06E-02 | 2,64E-02 | 2,46E-02 | 3,56E-02 | 1,23 | 0,332865 | 1,24 | 0,136702 | 1,35 | 0,177961 |
| **NFKBIA** | 3,79E-02 | 3,74E-02 | 3,42E-02 | 3,83E-02 | 6,65E-02 | 1,11 | 0,921743 | -1,02 | 0,935165 | 1,94 | 0,021750 |
| **NME1** | 3,07E-01 | 3,32E-01 | 1,88E-01 | 2,81E-01 | 3,73E-01 | 1,63 | 0,074445 | 1,18 | 0,535809 | 1,98 | 0,024148 |
| **NME4** | 1,92E-01 | 1,85E-01 | 1,93E-01 | 3,05E-01 | 4,37E-01 | -1,00 | 0,896154 | -1,65 | 0,111408 | **2,27** | **0,009719** |
| **PDGFA** | 1,41E-02 | 9,19E-03 | 1,12E-02 | 9,28E-03 | 3,37E-02 | 1,26 | 0,206187 | -1,01 | 0,997159 | **3,02** | **0,000014** |
| **PDGFB** | 4,00E-04 | 4,98E-04 | 1,66E-04 | 1,78E-04 | 2,89E-02 | 2,41 | 0,057864 | **2,80** | **0,027891** | **174,40** | **0,000107** |
| **PIK3R1** | 1,50E-02 | 1,38E-02 | 8,20E-03 | 1,28E-02 | 2,43E-02 | 1,83 | 0,030961 | 1,08 | 0,963487 | **2,97** | **0,001302** |
| **PLAU** | 7,22E-02 | 6,71E-02 | 1,22E-01 | 1,90E-01 | 4,73E-02 | -1,68 | 0,017562 | -2,84 | 0,060119 | **-2,57** | **0,003529** |
| **PLAUR** | 1,23E-01 | 1,08E-01 | 9,76E-02 | 1,71E-01 | 1,90E-01 | 1,27 | 0,344190 | -1,58 | 0,219587 | 1,94 | 0,040143 |
| **PNN** | 7,18E-02 | 6,07E-02 | 9,31E-02 | 1,18E-01 | 2,50E-01 | -1,30 | 0,074297 | -1,95 | 0,104885 | **2,69** | **0,008493** |
| **RAF1** | 4,57E-02 | 3,88E-02 | 3,51E-02 | 3,77E-02 | 5,67E-02 | 1,30 | 0,242324 | 1,03 | 0,847711 | 1,61 | 0,138478 |
| **RB1** | 5,12E-02 | 4,04E-02 | 3,85E-02 | 4,83E-02 | 5,69E-02 | 1,33 | 0,092092 | -1,19 | 0,463092 | 1,48 | 0,094764 |
| **S100A4** | 2,75E-03 | 2,96E-03 | 7,68E-03 | 5,09E-03 | 5,30E-03 | -2,80 | 0,111490 | -1,72 | 0,705158 | -1,45 | 0,350527 |
| **SERPINB5** | 7,16E-04 | 6,58E-04 | 1,66E-04 | 1,86E-04 | 6,70E-04 | 4,32 | 0,056954 | **3,54** | **0,010117** | **4,04** | **0,000712** |
| **SERPINE1** | 2,48E+00 | 2,05E+00 | 2,09E+00 | 3,21E+00 | 2,11E-01 | 1,19 | 0,502649 | -1,56 | 0,088266 | **-9,91** | **0,004158** |
| **SNCG** | 2,97E-03 | 2,62E-03 | 4,12E-03 | 2,24E-03 | 6,59E-03 | -1,39 | 0,374723 | 1,17 | 0,819064 | 1,60 | 0,218747 |
| **SYK** | 3,19E-04 | 3,83E-04 | 1,66E-04 | 2,01E-04 | 3,65E-04 | 1,92 | 0,207446 | 1,91 | 0,228455 | 2,20 | 0,073757 |
| **TEK** | 1,78E-02 | 3,18E-02 | 2,14E-02 | 6,12E-02 | 3,04E-03 | -1,20 | 0,619145 | -1,93 | 0,147586 | **-7,05** | **0,045652** |
| **TERT** | 2,43E-04 | 2,90E-04 | 1,66E-04 | 1,78E-04 | 1,50E-04 | 1,46 | 0,370941 | 1,63 | 0,165870 | -1,11 | 0,877564 |
| **TGFB1** | 2,56E-01 | 1,96E-01 | 2,23E-01 | 2,51E-01 | 2,78E-01 | 1,15 | 0,566388 | -1,28 | 0,400011 | 1,25 | 0,362738 |
| **TGFBR1** | 7,33E-03 | 5,03E-03 | 6,34E-03 | 4,84E-03 | 1,20E-03 | 1,16 | 0,804700 | 1,04 | 0,534841 | -5,27 | 0,176718 |
| **THBS1** | 3,23E+00 | 3,40E+00 | 2,50E+00 | 2,95E+00 | 1,74E-01 | 1,29 | 0,858153 | 1,15 | 0,721876 | -14,36 | 0,054108 |
| **TIMP1** | 3,32E+00 | 4,28E+00 | 3,09E+00 | 4,78E+00 | 3,03E+00 | 1,08 | 0,732198 | -1,12 | 0,514991 | -1,02 | 0,889734 |
| **TIMP3** | 7,86E-04 | 5,71E-04 | 1,66E-04 | 1,96E-04 | 2,33E-04 | 4,74 | 0,080303 | **2,91** | **0,012531** | 1,40 | 0,598055 |
| **TNF** | 7,53E-04 | 6,17E-04 | 1,86E-04 | 2,23E-04 | 2,87E-04 | **4,04** | **0,030749** | **2,77** | **0,010705** | 1,54 | 0,285973 |
| **TNFRSF10B** | 9,93E-02 | 1,01E-01 | 9,29E-02 | 1,81E-01 | 1,08E-01 | 1,07 | 0,929306 | -1,80 | 0,101515 | 1,17 | 0,569083 |
| **TNFRSF1A** | 1,21E-01 | 1,13E-01 | 1,18E-01 | 1,43E-01 | 7,67E-02 | 1,02 | 0,974093 | -1,27 | 0,283785 | -1,54 | 0,132799 |
| **TNFRSF25** | 5,36E-03 | 3,21E-03 | 7,38E-03 | 6,92E-03 | 5,65E-03 | -1,38 | 0,216637 | -2,15 | 0,092269 | -1,31 | 0,418065 |
| **TP53** | 1,06E-01 | 7,48E-02 | 9,77E-02 | 1,03E-01 | 1,65E-01 | 1,08 | 0,742575 | -1,38 | 0,300339 | 1,69 | 0,019120 |
| **TWIST1** | 8,58E-02 | 5,08E-02 | 5,75E-02 | 4,40E-02 | 1,71E-04 | 1,49 | 0,135781 | 1,15 | 0,908588 | **-336,33** | **0,003209** |
| **EPDR1** | 4,17E-02 | 4,21E-02 | 4,76E-02 | 4,29E-02 | 3,01E-02 | -1,14 | 0,523325 | -1,02 | 0,591534 | -1,58 | 0,036510 |
| **VEGFA** | 3,07E-01 | 9,12E-02 | 6,04E-01 | 6,24E-01 | 4,85E-02 | -1,97 | 0,006089 | **-6,84** | **0,008108** | **-12,44** | **0,000009** |
